# Dynamic Response of Heart Rate Variability to Active Standing in Aortic Valve Disease: Insights from Recurrence Quantification Analysis

**DOI:** 10.3390/s25051535

**Published:** 2025-03-01

**Authors:** Itayetzin Beurini Cruz-Vega, Nydia Ávila-Vanzzini, Gertrudis Hortensia González-Gómez, Rashidi Springall, Juan C. Echeverría, Claudia Lerma

**Affiliations:** 1 Department of Molecular Biology, Instituto Nacional de Cardiología Ignacio Chávez, Mexico City 14080, Mexico; ibcruzve@comunidad.unam.mx; 2 Plan de Estudios Combinados en Medicina, Faculty of Medicine, Universidad Nacional Autónoma de México, Mexico City 04510, Mexico; 3 Department of Outpatient Consult, Instituto Nacional de Cardiología Ignacio Chávez, Mexico City 14080, Mexico; anydia@medimanage.com.mx; 4 Department of Physics, Faculty of Sciences, Universidad Nacional Autónoma de México, Mexico City 04510, Mexico; hortecgg@ciencias.unam.mx; 5 Department of Immunology, Instituto Nacional de Cardiología Ignacio Chávez, Mexico City 14080, Mexico; maria.springall@cardiologia.org.mx; 6 Department of Electrical Engineering, Universidad Autónoma Metropolitana Unidad Iztapalapa, Mexico City 09340, Mexico; jcea@xanum.uam.mx

**Keywords:** cardiac autonomic modulation, aortosclerosis, aortic stenosis, orthostatic challenge, heart rate variability, recurrence plot analysis

## Abstract

Introduction: Aortic valve disease (AVD) is an inflammatory, lipid infiltration and calcification disease that has been associated with changes in the conventional linear heart rate variability (HRV) indices showing a marked shift towards sympathetic predominance and a deterioration of the autonomic control. Objective: To explore the HRV dynamics in AVD patients through nonlinear methods by recurrence quantification analysis (RQA). Methods: In total, 127 subjects participated in a cross-sectional study categorized into three groups: healthy valve (HV), aortic valve sclerosis (AVSc), and aortic valve stenosis (AVS), as determined by echocardiographic assessment. HRV data were collected from five-minute ECG recordings at both a supine position and active standing. RQA indices were calculated using the Cross Recurrence Plot Toolbox. Results: In the supine position, patients with AVS exhibited larger determinism and trapping time than those with AVSc and HV. The analysis of these differences revealed that determinism and laminarity increased progressively from HV to AVS. In the same way, the magnitude of change (Δ) between positions decreased and presented the lowest values in AVS in most of the nonlinear indices. Conclusion: RQA indices of HRV in AVD patients indicate a rigidizing dynamic characterized by larger determinism and extended trapping times in fewer system states in relation to the severity of AVD. These findings establish a precedent for future perspective assessments for the implementation of these methods in medical software or devices.

## 1. Introduction

### 1.1. Epidemiology of AVD

Aortic valve disease (AVD) is on the rise due to an aging population, with aortic stenosis (AS) being the most prevalent form [1]. The incidence case of AVD gradually increased by 351% from 130,821 in 1990 to 589,637 cases in 2019; meanwhile, the age-standardized incidence rate (ASIR) increased by 120% from 3.25 in 1990 to 7.13 per 100,000 in 2019. Globally, the disability-adjusted life years (DALYs) of AVD increased by 88.3% from 975,894 in 1990 to 1,837,751 in 2019, leading to a significant increase in morbidity and mortality [2,3,4].

### 1.2. Pathophysiology of AVD

Aortic stenosis (AS) develops due to an inflammatory process initiated by endothelial damage resulting from mechanical stress and lipid infiltration, ultimately causing leaflet thickening and calcification [4]. While aortic valve sclerosis (AVSc) represents an early stage of AVD that does not exhibit structural changes or hemodynamic consequences, up to 10–15% of patients with AVSc may progress to significant valve obstruction within 2 to 5 years, thereby increasing their risk of cardiovascular complications [5]. Risk factors such as hypertension, diabetes, and dyslipidemia significantly increase the likelihood of developing severe AS, contributing to about one-third of cases in older populations [4,6,7]. Unfortunately, current treatments, including antihypertensive agents and statins, do not halt the disease’s progression, and aortic valve replacement is the only intervention that has been demonstrated to improve survival [8,9,10,11,12,13].

### 1.3. Heart Rate Variability (HRV)

The autonomic nervous system (ANS) regulates the interplay between sympathetic and parasympathetic systemic responses, enabling the adaptation to physiological stimuli, such as postural changes. The orthostatic challenge (performed by changing the supine position to active standing) has been used as a proxy study of autonomic cardiac modulation by assessing heart rate variability (HRV), which is also referred to as the interval fluctuations between consecutive heartbeats [14,15]. Linear methods provide valuable approximations of feedback mechanisms within the ANS. Linear approaches, providing statistical and spectral parameters, enable the estimation of sympathetic and parasympathetic contributions of the ANS. In contrast, although less explored, nonlinear methods, such as recurrence plots analysis, allow for the characterization of the dynamic processes underlying cardiovascular adaptability to various stimuli [16].

#### 1.3.1. Linear Analysis of HRV

In healthy subjects, the linear analysis of HRV reveals that parasympathetic influences predominantly drive the supine position, while active standing is characterized by reduced parasympathetic activity and increased sympathetic response, reflected as a decrease in HRV. But even when supine, as AVD progresses from AVSc to AS, there is a shift towards sympathetic predominance. Previous studies have shown that severe AS is associated with reductions in several HRV indices, indicating impaired autonomic control likely due to vagal withdrawal and heightened sympathetic activity [15,17,18,19,20,21]. Importantly, all linear HRV indices depend on the mean heart rate, a relationship observed in healthy individuals and those with certain diseases such as chronic renal disease [22,23], which also changes by the AVD progression [15,20].

#### 1.3.2. Nonlinear Analysis of HRV

Nonlinear methods for heart rate variability (HRV) analysis are used to capture the dynamic complexity of the cardiovascular system, which originates from multiple interacting regulatory processes and cannot be adequately described by linear methods alone. Among these methods, scaling exponents [15] and Shannon entropy [24] have been evaluated in AVD. However, unlike these approaches, recurrence quantification offers greater robustness and sensitivity, particularly for analyzing short and non-stationary time series [25]. In the review of the literature, this method is addressed for the description of the dynamics of HRV; for instance, in healthy subjects, RQA indices indicate an increment in determinism and laminarity, signifying a more periodic dynamic behavior of HRV with fewer dynamic states and more stability at active standing [14,24,26]. Similar changes in HRV dynamics have been observed in patients with conditions that disrupt the sympathovagal balance, such as diabetes and chronic kidney disease [14,22,26]. These insights are essential for early diagnosis and developing targeted therapeutic strategies, ultimately improving patient outcomes.

As already mentioned, no studies have explored RQA HRV indices, as autonomic cardiac modulation dynamics markers across the AVD spectrum in particular. Therefore, this study aims to explore RQA HRV dynamics and evaluate the correlation between RQA indices and the mean heart rate in participants with healthy valves, aortic valve sclerosis, and aortic valve stenosis during an orthostatic challenge.

## 2. Materials and Methods

### 2.1. Study Design and Patients

Overall, 127 volunteers participated in a cross-sectional study at the National Institute of Cardiology Ignacio Chavez in Mexico City, recruited from January 2017 to July 2023 using consecutive sampling. Eligibility criteria stipulated that participants must not have any ischemic, renal, inflammatory, or autoimmune diseases that could have interfered with the autonomic nervous system modulation. Additionally, participants were excluded if they were taking β-blockers or had any cardiac electrical conduction disorder diagnosed with a 12-lead electrocardiogram evaluation examined by a cardiologist.

Out of 98 presumably healthy volunteers who underwent a routine clinical evaluation that included an echocardiogram to rule out the presence of any disease and comorbidities, only 22 individuals were found to have no structural or hemodynamic alterations of the aortic valve and were assigned to the healthy valve group. In contrast, 73 subjects exhibited calcified focal areas with increased echogenicity and thickened aortic valve leaflets, and were classified into the aortic valve sclerosis group.

Additionally, 47 patients from the Institute’s valvular disease clinic, previously diagnosed with aortic valve stenosis, were also invited to participate. Eight had another heart valve condition, and six had an etiological cardiac malformation. Ultimately, the included group of patients with aortic valve stenosis consisted of 32 individuals. Exclusion criteria included the presence of supraventricular arrhythmias, identified by ECG artifacts and assessed by blinded experts.

Informed consent was obtained from all participants following the ethical standards established by the Research and Ethics Committee of the National Institute of Cardiology Ignacio Chávez (protocols 16-993 and 18-1090) and the Helsinki Declaration.

### 2.2. Study Protocol

A comprehensive medical history was obtained for all participants, emphasizing AVD risk factors such as smoking and alcohol use and relevant comorbidities, including hypertension, type II diabetes, and dyslipidemia. Laboratory tests were performed by collecting a 10 mL blood sample (with 8 h minimum of fasting) from each participant. All samples were centrifuged at 3000 rpm for 15 min. Metabolic parameters were reported by blood chemistry: cholesterol (mg/dL), C-HDL (mg/dL), C-LDL (mg/dL), triglycerides (mg/dL), and atherogenic index.

The dynamic variables of the aortic valve stenosis were obtained by conventional transthoracic echocardiograms (iE33, Philips Healthcare, Bothell, WA, USA) and pulsed-wave Doppler recordings (performed by a cardiologist from our group with more than 10 years of experience) to assess maximum aortic valve transvalvular velocity (m/s, Vmax), mean pressure gradient (mmHg, mean gradient), aortic valve area (cm^2^, AVA), and left ventricular ejection fraction (%, LVEF). All measurements corresponded to the average of three consecutive assessments of each parameter throughout cardiac cycles. All patients were categorized into three different groups: healthy valve (HV); aortic valve sclerosis (AVSc) defined by calcified focal areas of increased echogenicity and thickened aortic-valve leaflets with no motion restriction or obstruction of the left ventricular outflow tract and antegrade flow evidenced in a pulsed-wave Doppler recording [6]; and aortic valve stenosis (AVS) according to the following echocardiograms parameters: AVA < 1.5 cm^2^, Vmax > 3 m/s, and mean pressure gradient > 25 mmHg [27].

### 2.3. Electrocardiogram Recording and Beat Identification

Ten minutes of ECG data in each position were collected according to the validated orthostatic challenge protocol (supine and active standing) [14,15,18,20,22,26] with a chest belt (BioHarness 3.0, Zephyr Technology, Annapolis, MD, USA) at a sampling rate of 1000 samples per second. Data collection started when the patient was in a supine position and ended 10 min after changing to active standing. The initial minutes were excluded to ensure hemodynamic stabilization, and thus the final five-minute segment from each position was selected. The duration and sampling rate followed the Task Force guidelines, which recommend a minimum sample rate of 250 Hz [28]. The QRS complexes were detected using a second derivative algorithm [29]. Subsequent manual inspection was conducted to eliminate artifacts and ectopic beats, ensuring that no more than 5% of beats required replacement using adaptive filtering [30], resulting in a heart rate variability (HRV) time series.

### 2.4. Heart Rate Variability Analysis

#### 2.4.1. Time Domain and Frequency Domain

HRV time-domain indices were computed for each time series, including mean NN (average value of all RR intervals originated in normal-sinus-rhythm), SDNN (standard deviation of all RR intervals), pNN20 (percentage of successive RR intervals with differences greater than 20 ms), and SDSD (the standard deviation of the successive differences in NN intervals) [28]. Each time series was resampled for estimating HRV frequency domain indices using a linear interpolation at three samples per second, and the power spectral density was estimated using Welch’s periodogram method. The mean spectral power was obtained for the low frequency (LF) band (0.04 to 0.15 Hz) and the high frequency (HF) band (0.15 to 0.4 Hz). LF and HF indices were expressed in normalized units (n.u.) [29]. While LF reflects the activities of both the sympathetic and parasympathetic systems, HF is considered as a reliable indicator of vagal cardiac control. These indices indicate the irregularity and directionality of time series, which are influenced by the cardiac-autonomic interplay [31]. The difference (∆) in all HRV indices between the supine position and active standing was also calculated. HRV indices were estimated using our custom and validated programs developed with MATLAB software (version R2018a; MathWorks, Inc., Natick, MA, USA)

#### 2.4.2. Recurrence Plot Analysis

The time-delay embedding method was applied to reconstruct RR interval data in a multidimensional state space. Each point in the reconstructed phase space represents the system’s state at a specific time, determined by the m coordinates corresponding to a given embedding dimension [25]. To obtain these reconstructed data, we used the tools developed by Norbert Marwan and colleagues: Cross Recurrence Plot Toolbox for MATLAB (available from the toolbox for complex systems (TOCSY) in https://tocsy.pik-potsdam.de/CRPtoolbox, accessed on 28 February 2025).

The embedding time delay for each RR time series was estimated by its autocorrelation function [26]. The average autocorrelation from all recordings dropped to zero right before the time delay = 6. Therefore, the embedding time delay was set to 6 for all recordings. The embedding dimension (m) for each recording was estimated using the false nearest neighbor’s method. The average percentage of false neighbors from all recordings dropped to 10% at the embedding dimension = 5. Therefore, we chose m = 5 as the embedding dimension for all series [14]. Subsequently, distances between individual points in the matrix, representing the system’s state at a given time, were calculated using the Maximum Norm−Fixed Recurrence Rate [32].

The most commonly used neighborhood is that with a fixed radius [33] that produces a symmetric RP. However, in the original definition of the RPs, the neighborhood is a ball, and its radius is chosen to contain a fixed number of states [34], the radius changes for each state. This neighborhood is denoted as a fixed number of nearest neighbors (FAN). In our context of cardiovascular signals recordings, the distance threshold is justified not using a fixed value due to the considerable inter- and intraindividual variability. That fixes the number of recurrent points for each vector, i.e., its density.

We evaluated the following most commonly used indices related to recurrence quantification analysis (RQA) [14,15,18,20,22,26] through the assessment of more than one diagonal and vertical parameter on the dynamics of HRV: determinism (percentage of recurring points that form diagonal lines in the phase space); mean diagonal length; maximum diagonal length; laminarity (proportion of recurrence points forming vertical lines); maximum vertical length; trapping time (time in which the dynamics remain trapped in a particular state); maximal length of the vertical lines; recurrence time type 1; and recurrence time type 2. The recurrence types are determined according to the plot’s vertical distance between recurrence points [35]. Additionally, entropy was calculated as Shannon information entropy derived from the distribution of line lengths, indicating the amount of information required to determine the system’s state. Notably, all assessed RQA indices exhibited similar behavior across varying recurrence rates, with significant and consistent increases observed in both supine and active standing positions as the recurrence rate increased [26].

The recurrence plot is the graph of a square matrix in which the matrix elements represent the times at which the state of a dynamical system recurs, revealing the times when the phase space trajectory of the dynamical system visits the same area (i.e., neighborhood) in the phase space [25]. There are several variations of recurrence plots. As examples, there are the recurrence plots based on the definition of the neighborhood with a fixed radius [34] or those from a changing radius that adjusts to the density of recurrence points to ensure a fixed number of states (i.e., fixed number of neighbors) [25]. Another variation is the perpendicular recurrence plot, which includes only the points lying on perpendicular trajectories in the phase space [36]. These and other variants are detailed at http://www.recurrence-plot.tk/variations.php, accessed on 28 February 2025 [25]. The selection of recurrence plot variants depends on the problem studied and the type of data. We selected the recurrence plot as defined by Marwan et al. [25] with a fixed number of neighbors based on our experience with short-term HRV time series in healthy populations [37] and patients with different pathologies [38].

### 2.5. Statistical Analysis

Nominal variables were reported as absolute values (percentages) and compared between groups using the Chi-squared and Fisher’s exact tests. The Kolmogorov–Smirnov test for normal distributions was applied for ordinal and continuous variables. Results are expressed as mean ± standard deviation and compared between groups (HV vs. AVSc vs. AVS) using an ANOVA for repeated measures, or these results are expressed as median (25th percentile–75th percentile) and compared using the Kruskal–Wallis test followed by Mann–Whitney U test and the Wilcoxon test. The magnitude of change (Δ) of each index in response to active standing was calculated as the difference between supine position—active standing. A Wilcoxon test was applied to test significant changes in response to active standing (i.e., the median of Δ was different from zero).

A Pearson correlation analysis assessed the relationship between mean NN and HRV indices. Logistic regression analysis was also executed to evaluate the association between mean NN and selected variables representative of AVD conditions (presence of AVS), age, and systolic blood pressure (SBP). A value of *p* < 0.05 was considered statistically significant. Missing data were excluded from analyses, so these were not replaced. Statistical analyses were performed using SPSS version 21.0 (IBM Corp., Armonk, NY, USA).

## 3. Results

### 3.1. Demographic Data

The study included 127 patients in three groups: healthy valve (HV, N = 22), aortic valve sclerosis (AVSc, N = 73), and aortic valve stenosis (AVS, N = 32). In Table 1 we present the clinical and demographic characteristics of the patients, along with identified risk factors across the respective groups. The AVS patients were significantly older and exhibited higher prevalences of hypertension, type II diabetes, or dyslipidemia, and higher use of prescribed medication (statins and acetylsalicylic acid). Echocardiographic parameters indicative of aortic valve disease severity revealed smaller aortic valve area (AVA) and reduced left ventricular ejection fraction (LVEF), alongside greater peak velocity (Vmax), increased mean gradient, and elevated systolic blood pressure (SBP) in the AVS group compared to the other groups. There were no statistical differences in the other variables.

**Figure 1:**
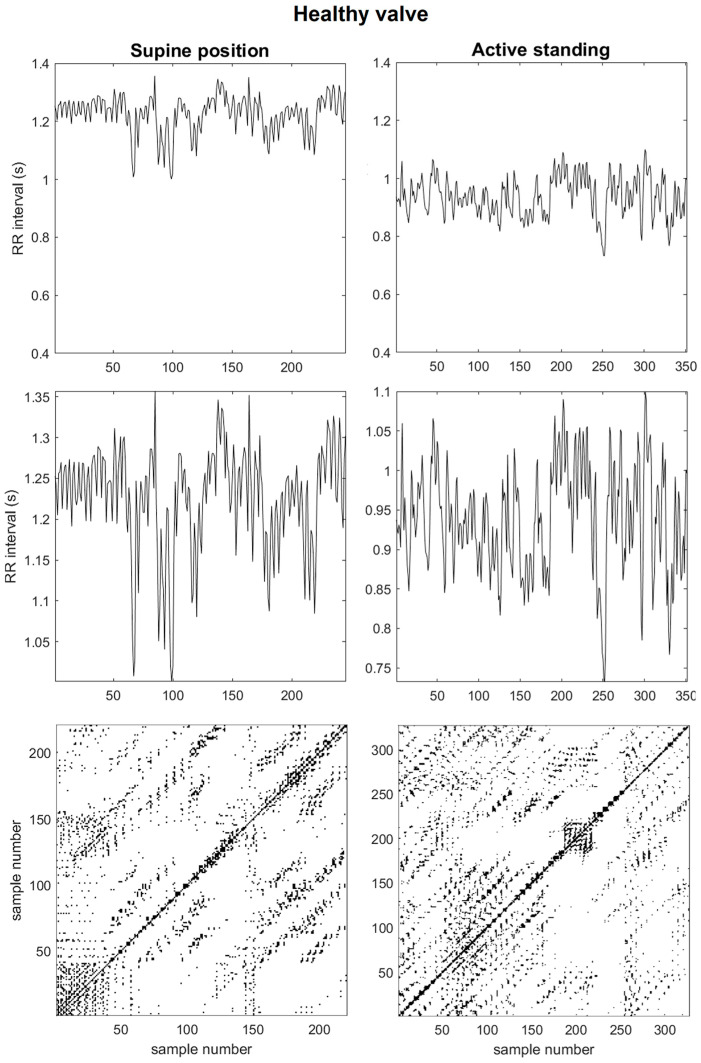
A representative case of a subject with healthy valve: The upper panel displays the RR time series for both positions. In the middle panel, enlarged views of the upper boxes to visualize the time series in greater detail are exemplified. At the bottom, the time series reconstruction is presented alongside the corresponding recurrence plots. RQA time-delay = 6 and time-dimension = 5.

Figure 1 shows a representative case of the healthy valve group. On visual inspection, a decrease in the variability of RR time series demonstrates the increase in heart rate in response to the active standing position compared to supine position. Regarding recurrence plots, in the supine position, the plot shows extended diagonal lines creating a regularly spaced pattern along the surface. In contrast, in the standing position, the white areas increase, indicating a reduction in the accessible dynamic states within the central region.

**Figure 2:**
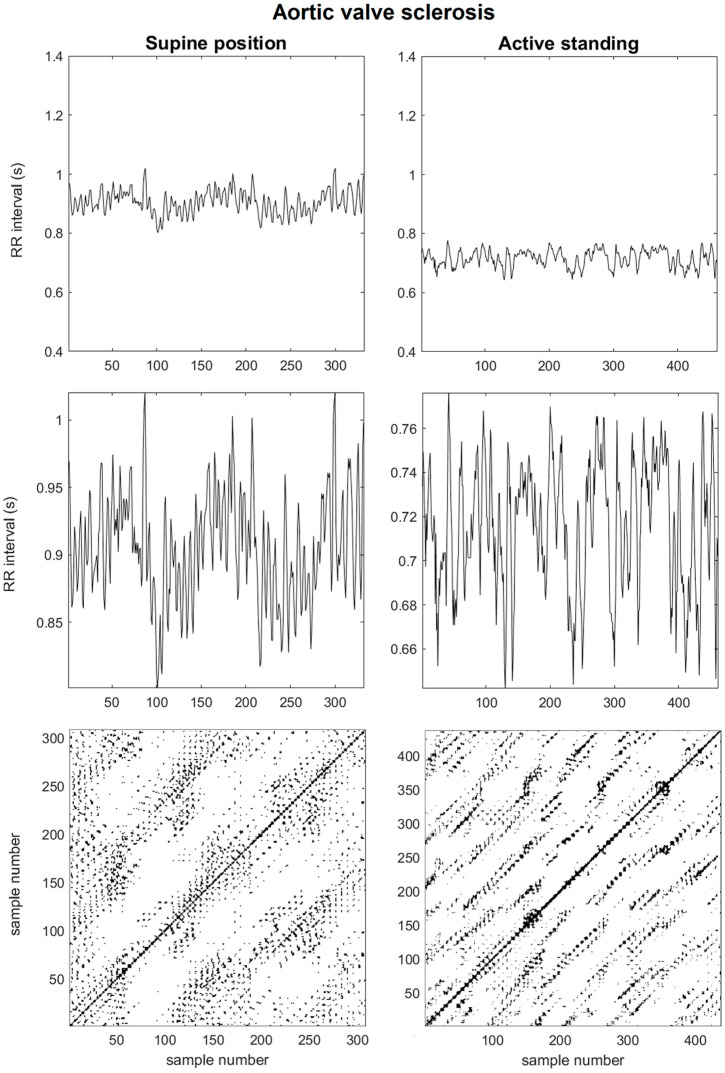
A representative case of a subject with aortic valve sclerosis: The upper panel displays the RR time series for both positions. In the middle panel, enlarged views of the upper boxes to visualize the time series in greater detail are exemplified. At the bottom, the time series reconstruction is presented alongside the corresponding recurrence plots. RQA time-delay = 6 and time-dimension = 5.

The representative case of the aortic valve sclerosis group, as depicted in Figure 2, exhibits significant differences compared to the healthy valve group. This example demonstrates a shorter RR interval, which suggests an elevated heart rate even from the baseline supine position. Upon transitioning to the orthostatic challenge, there is an increase from the baseline values. However, the magnitude of this change is lower than that observed in the healthy valve group. A closer examination of the upper frames reveals a dominance of high-frequency components during active standing. The recurrence plots for this group further highlight these changes, showing shorter diagonal lines in the supine position, which shift to longer diagonal lines in the orthostatic position. Additionally, larger white spaces appear across the map, reflecting the persistence of longer trajectories and a reduction of dynamic states.

**Figure 3:**
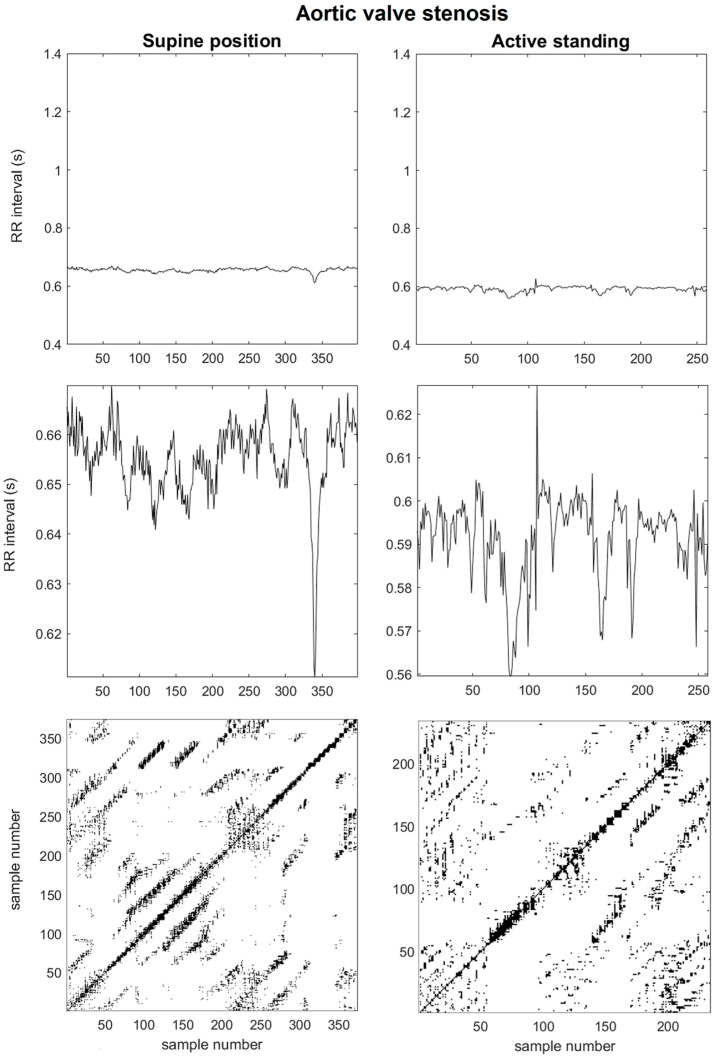
A representative case of a subject with aortic valve stenosis: The upper panel displays the RR time series for both positions. In the middle panel, enlarged views of the upper boxes to visualize the time series in greater detail are exemplified. At the bottom, the time series reconstruction is presented alongside the corresponding recurrence plots. RQA time-delay = 6 and time-dimension = 5.

In Figure 3, visual inspection of the HRV dynamics in a patient from the AVS group reveals a notorious decrease in the time series variability when compared to variability shown by healthy individuals. Although the RR interval decreases in response to active standing, the change is smaller than the examples of HV (Figure 1) and AVSc (Figure 2). In terms of the HRV dynamics, the presence of prominent diagonal lines, accompanied by vertical lines, indicates the persistence of certain states over time. In the active standing position, vertical lines predominately extend towards the periphery, reflecting a progressive increase in white spaces and a dynamical restriction of the traversed phase space.

### 3.2. HRV Linear Indices

In Table 2 we show the results of HRV linear indices related to the orthostatic challenge positions. Significant differences in mean NN intervals were observed between positions (supine vs. active standing) for each of the three groups. Moreover, Δ Mean NN decreased progressively with disease progression, showing lower values in AVS patients. However, no differences between groups were noted at the same position. In the supine position, AVS patients showed both decreased pNN20 and high-frequency normalized units (HF n.u), and both increased low-frequency normalized units (LF n.u) as well as LF/HF ratios compared to HV. A significant decrease in pNN20 was also noted in AVS patients compared to the AVSc group. During active standing, AVS patients exhibited reductions in SDNN and SDSD compared to HV. According to the Δ parameters between position, ΔHFn.u and ΔpNN20 decreased along the AVD spectrum, while ΔLFn.u and ΔLF/HF ratio increased, with higher values observed in the AVS group.

### 3.3. HRV Nonlinear Indices

**Figure 4:**
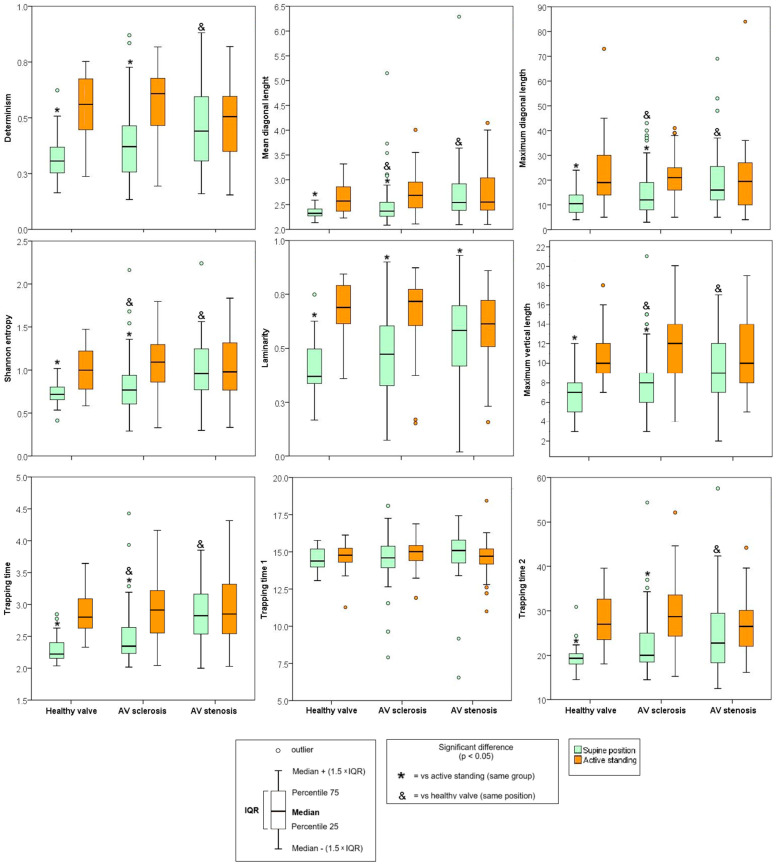
Results of the analysis of recurrence rates of heart rate variability. Data are shown as median (25th percentile–75th percentile). Determinism = the percentage of recurrence points forming diagonals from all recurrence points. Shannon entropy = related to the uncertainty of finding a diagonal line. Laminarity = proportion of recurrence points forming vertical lines. Trapping time = time in which the dynamics remain trapped in a certain state. A table is available in Supplementary Material File S1 with full details.

For HRV nonlinear indices during the orthostatic challenge, AVS patients in the supine position showed increased values of determinism, mean diagonal length, maximum diagonal length, Shannon entropy, trapping time, Vmax, and trapping time 1. No differences in laminarity and trapping time 2 were found compared to HV subjects. Additionally, increases in the mean diagonal length, maximum diagonal length, Shannon entropy, trapping time, and maximum vertical length were observed with no differences in determinism, laminarity, trapping time 1, and trapping time 2 compared to the AVSc group. Laminarity decreased in AVS patients during active standing compared to AVSc, while the remaining variables did not show significant differences (Figure 4).

**Figure 5:**
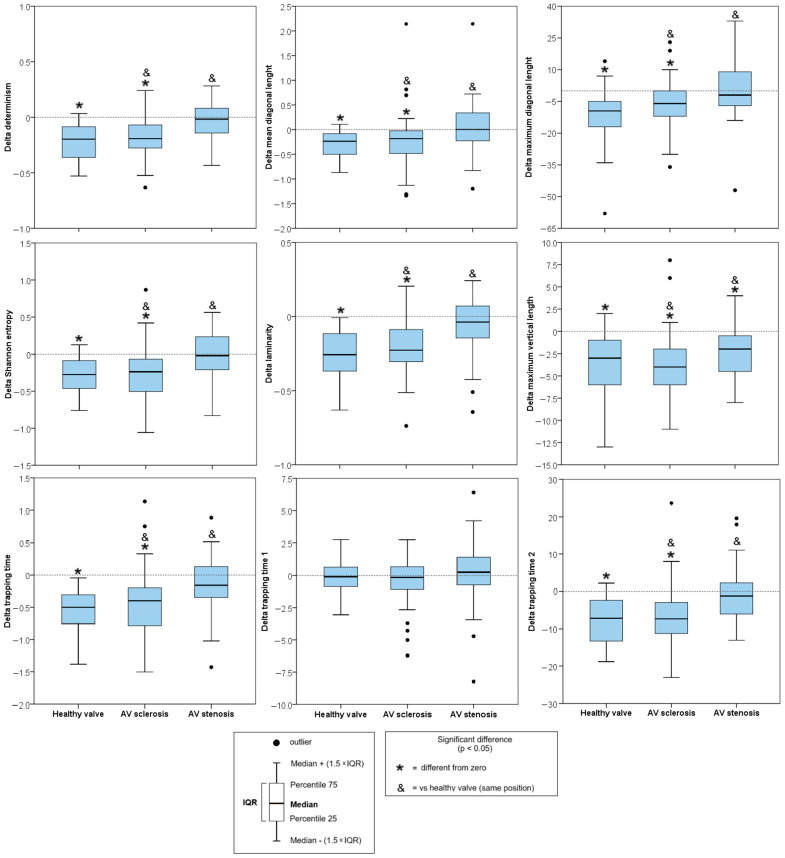
Results of the analysis of magnitude of change (Δ) of recurrence rates of HRV. Data are shown as median (25th percentile–75th percentile). Δ = difference between the values in supine position and values in active standing for in each HRV index. A table is available in Supplementary Material File S1 with full details.

In terms of Δ parameters between RQA parameters, Δ determinism, Δ mean diagonal length, Δ maximum diagonal length, Δ Shannon entropy, Δ laminarity, Δ trapping time, and Δ trapping time 2 increased in AVSc and the in AVS (Figure 5).

### 3.4. Correlations Between meanNN and HRV Indices

Correlations between meanNN and HRV indices are shown in Table 3. Significant differences were found in all variables except for the correlation with LF/HF in AVS patients. Interestingly, most variables (SDNN, pNN20, SDSD, and HF) displayed lower correlation power values between HV and AVS, while LF n.u showed increased correlation power values.

Table 4 summarizes the correlations between mean NN intervals and HRV nonlinear indices, revealing significant correlations for all variables, except for T1 in AVSc and AVS patients. The remaining variables exhibited higher correlation power values between HV and AVS.

### 3.5. Multiple Regression Analyses and HRV Indices

Further linear stepwise multiple regression analyses were conducted, considering the mean NN, age, SBP, statins, and AVS condition as the independent variables and the HRV indices as the predicted variables (Table 5 and Table 6). The association of mean NN with all indices was evident, particularly with age and SBP, especially in the HRV nonlinear analyses.

## 4. Discussion

The main contribution of this work is that nonlinear HRV indices, assessed by recurrence quantification analysis, demonstrated significant differences between patients with AVD compared to subjects with healthy valves, as summarized in Table 7. In response to the orthostatic challenge, most RQA indices, with the exception of trapping time 1, showed increments in active standing in both the HV and AVSc groups. In contrast, the AVS group exhibited only increases in laminarity and maximum vertical length. When comparing the groups to the HV group, the AVSc group displayed increases in mean diagonal length, maximum diagonal length, Shannon entropy, trapping time, and maximum vertical length. In the AVS group, these same indices, along with determinism and trapping time 2, were also elevated. This shift suggests a reduction in the exploration of states within the time series and shorter recurrence points in a three-dimensional reconstruction, indicating a transition toward more rigid heart rate behavior. These changes reflect a decrease in the complexity and predictability of heart rate dynamics.

These findings are further supported by the observed decreases in most Δ changes (Δ determinism, Δ mean diagonal length, Δ maximum diagonal length, Δ Shannon entropy, Δ laminarity, Δ trapping time, and Δ trapping time 2) across the AVD groups (AVSc and AVS) compared to the HV group. The statistical test results further revealed that the Δ values of the indices are significantly different from zero in the HV and AVSc groups. In contrast, the AVS group shows differences only in the maximum vertical length index, confirming the absence of significant differences between positions and thus indicating limited involvement of adaptability process in response to this orthostatic challenge (Table 8).

Regression analyses further support the significance of these indices, showing associations with age, systolic blood pressure, and statins, especially in the HRV nonlinear indices. This could be explained by the age difference between the groups, considering the natural history of the disease and the age groups most frequently affected by this condition. In the case of statins, the association can be explained by the fact that these medications are indicated for their lipid-lowering influence and also, therefore, anti-inflammatory effect on AVD, which seems to be required owing to the chronic inflammatory process of this disease. On the other hand, the dichotomic (categorical) variable AVS condition (HV vs. AVD) was not significant as a predictor within the multiple regression model.

The results are in accordance with prior research highlighting the complexity of heart rate dynamics in advanced aortic stenosis. For instance, Ackun (2024) noted that nonlinear indices, such as entropy, reveal significant alterations in heart rate dynamics, reflecting intricate interactions between the autonomic nervous system and the cardiovascular system in severe cases of aortic stenosis [24]. Moreover, here, RQA parameters were higher in patients with aortic valve disease (AVD) than in healthy individuals, indicating that AVD subjects exhibit lower activity levels, with similar signal patterns occurring more frequently in coincidence with another study of coronary artery disease patients [39]. These findings, which could be associated with an increased sympathetic regulation may result from adaptive changes throughout the AVD spectrum. Hence, applying RQA indices to AVSc patients could enhance monitoring disease progression and treatment responses. Nonlinear HRV measures have been associated with disease severity and functional status across various cardiovascular conditions, including AVSc [24].

Regarding linear HRV indices, in our patients with AVS, pNN20, HFn.u, and SDNN exhibited a marked decrease at the orthostatic challenge positions compared to those obtained from subjects with healthy valves. This decrease reflects impaired parasympathetic activity and overall autonomic dysfunction. The progressive decline in ΔMean NN and other linear indices as the disease advances from AVSc to AVS further indicates deteriorating autonomic control. This suggests that linear HRV indices may be markers of the disease progression and autonomic deterioration in AVD.

A previous study involving 61 participants—16 without aortic valve sclerosis (NAVS) and 45 with AVS—found a significant difference in the low-frequency (LF) to high-frequency (HF) ratio during the supine position (Ln(LF/HF) = 0.85 ± 0.85 vs. 0.11 ± 0.69, *p* = 0.003). This indicates a significant reduction in parasympathetic cardiac modulation in AVS patients compared to healthy valves. Additionally, during orthostatic challenges, AVS patients exhibited diminished sympathetic adjustments [15]. The observed decrease found here in linear HRV indices such as pNN20, HFn.u, and SDNN in AVS patients at the orthostatic challenge is consistent with previous findings of reduced parasympathetic activity, which coincides with our study’s observation. This relationship underscores the progression of autonomic dysfunction in AVD, aligning with broader patterns reported in the literature [20].

The prevalence of comorbidities, particularly hypertension and type II diabetes, is higher in the AVS group compared to the other groups. The difference in hypertension prevalence can be attributed to the underlying pathophysiology of the disease, where adaptive changes in ventricular myocardial remodeling and baroreceptor-mediated pressure regulation contribute to this physiological response [5,11,17,21,40,41]. In contrast, type II diabetes, driven by a pathological inflammatory process, may influence cardiac autonomic modulation through the cholinergic anti-inflammatory pathway [42], potentially affecting HRV dynamics. This should be considered when interpreting the results as it represents a significant study limitation.

Incorporating these advanced analytical techniques into routine clinical practice could allow clinicians to better understand the autonomic changes associated with AVD, facilitating more effective treatment adjustments. This approach could lead to personalized management strategies and improved prognostic assessments, ultimately improving patient care and therapeutic outcomes. Although the technological infrastructure necessary for implementing these new analysis methods in software, as well as portable and wearable devices, is available, enabling the development of clinical tools for identifying high-risk patients, several methodological challenges remain. Despite providing valuable insights into the complexities of underlying physiological mechanisms, these methods are hindered by the operator dependence in setting the parameters, thereby presenting a significant challenge for future implementations.

Despite the valuable insights from this study, there were several limitations. The small sample size, particularly within the AVS group, may affect the generalization of our results. Furthermore, the cross-sectional design limits the ability to establish causal relationships or to observe longitudinal HRV changes throughout disease progression. Future research could address these limitations by employing larger, longitudinal studies to deepen the understanding of autonomic dysfunction within the AVD spectrum and enhance its clinical applicability.

## 5. Conclusions

In conclusion, this study highlights the significant alterations in heart rate variability (HRV) dynamics across the aortic valve disease (AVD) spectrum based on linear HRV and RQA indices. Our findings indicate that as AVD progresses from aortic valve sclerosis to aortic stenosis, there is a marked shift towards sympathetic predominance and a deterioration of the autonomic control, reflected by linear HRV indices. Furthermore, HRV nonlinear indices describe a transition to a more rigid heart rate behavior and reduced variability as the disease progresses. These insights underscore the potential of RQA indices to provide a more nuanced assessment of autonomic dysfunction and cardiovascular risk, offering valuable tools for early diagnosis and targeted therapeutic strategies. By enhancing our understanding of the autonomic changes associated with AVD, this research may contribute to developing more personalized management approaches, ultimately improving clinical outcomes in this growing population of patients with AVD.

## Figures and Tables

**Table 1:** Clinical and demographic characteristics of study participants. Data are shown as mean ± standard deviation, median (25th percentile–75th percentile), or absolute value (percentage).

Variables	Healthy Valve (N = 22)	Aortic Valve Sclerosis (N = 73)	Aortic Valve Stenosis (N = 32)	*p*
Age (years)	41.3 ± 7.9	45.3 ± 9.3	63.3 ± 6.6	<0.001
Gender (Men)	12 (54.5)	33 (45.2)	21 (65.6)	0.151
**Metabolic variables**				
BMI (kg/m^2^)	27.35 ± 3.69	28.16 ± 4.72	29.50 ± 4.94	0.210
Hypertension	2 (3.8)	4 (12.6)	16 (50)	<0.001
Diabetes type II	0	2 (2.7)	7 (21.9)	0.002
Dyslipidemia	0	5 (6.8)	10 (31.3)	0.001
Alcoholism	10 (45.5)	32 (43.8)	17 (53.1)	0.676
Smoking	6 (27.3)	26 (35.6)	12 (37.5)	0.714
Statins	0	0	7 (5.5)	<0.001
Acetylsalicylic acid	0	0	10 (31.3)	<0.001
Cholesterol total (mg/dL)	192 ± 34	192 ± 38	177 ± 37	0.150
C-HDL (mg/dL)	41.59 ± 10.54	44.31 ± 8.55	43.17 ± 10.75	0.485
C-LDL (mg/dL)	125 ± 32	124 ± 33	103 ± 33	0.009
Triglycerides (mg/dL)	139 (113–163)	133 (94–188)	144 (102–188)	0.927
Atherogenic index	3.21 ± 1.19	2.88 ± 0.86	2.58 ± 1.23	0.082
**Echocardiographic parameters**				
Vmax (m/s)	1.2 (1.0–1.3)	1.3 (1.1–1.4)	4.23 (3.2–5.5)	<0.001
Mean gradient (mmHg)	3.0 (2.0–3.0)	3.0 (2.0–4.0)	41.0 (23.0–71.0)	<0.001
LVEF (%)	61.9 ± 6.4	62.3 ± 6.6	54.7 ± 8.6	<0.001
Heart rate (beats per minute)	60.8 ± 9.7	60.9 ± 8.7	63.0 ± 10.8	0.549
AVA (cm^2^)	4.2 (4.03–4.2)	4.1 (3.96–4.26)	0.6 (0.41–1.26)	<0.001
SBP (mmHg)	110 (110–118)	116 (110–122)	130 (120–160)	<0.001
DBP (mmHg)	78.0 (70.0–80.0)	78.0 (70.0–80.0)	80.0 (70.0–80.0)	0.478

BMI = Body Mass Index. Vmax = Maximum Aortic Valve Transvalvular Velocity. LVEF = Left ventricular Ejection Fraction. AVA = Aortic Valve Area. SBP = Systolic Blood Pressure. DBP = Diastolic Blood Pressure.

**Table 2:** Results of time domain and frequency domain analysis of heart rate variability. Data are shown as mean ± standard deviation.

	Healthy Valve (N = 22)	Aortic Valve Sclerosis (N = 73)	Aortic Valve Stenosis (N = 32)
**Supine position**			
Mean NN (s)	0.995 ± 0.180 **	0.991 ± 0.137 **	0.988 ± 0.159 **
SDNN (s)	0.051 ± 0.019	0.054 ± 0.025 *	0.045 ± 0.024
pNN20 (%)	57.4 ± 17.5 **	55.35 ± 19.39 **	40.37 ± 24.89 * b,c
SDSD (s)	0.041 ± 0.021 *	0.038 ± 0.020 **	0.029 ± 0.017 *
LF n.u	58.51 ± 15.66 **	65.14 ± 19.36 **	73.54 ± 28.17 b
HF n.u	44.06 ± 14.46 **	37.90 ± 17.96 **	31.42 ± 20.09 * b
LF/HF	1.57 ± 0.870 *	2.91 ± 4.03 **	4.92 ± 7.08 b
**Active Standing**			
Mean NN (s)	0.823 ± 0.153	0.835 ± 0.102	0.887 ± 0.121
SDNN (s)	0.053 ± 0.030	0.045 ± 0.013	0.039 ± 0.016 b
pNN20 (%)	39.28 ± 21.73	34.90 ± 18.07	30.10 ± 18.66
SDSD (s)	0.031 ± 0.024	0.023 ± 0.010	0.021 ± 0.009 b
LF n.u	80.74 ± 10.27	81.28 ± 11.96	75.79 ± 15.71
HF n.u	19.25 ± 10.27	18.71 ± 11.96	24.20 ± 15.71
LF/HF	5.61 ± 3.30	6.76 ± 5.25	4.59 ± 2.99
**Magnitude of change**			
Δ Mean NN (s)	0.171 ± 0.068	0.156 ± 0.081	0.100 ± 0.097 b,c
Δ SDNN (s)	−0.001 ± 0.024	0.009 ± 0.025	0.006 ± 0.020
Δ pNN20 (%)	18.128 ± 13.401	20.445 ± 16.450	10.274 ± 19.377 c
Δ SDSD	0.010 ± 0.011	0.015 ± 0.017	0.008 ± 0.015
Δ LF (n.u)	−22.235 ± 19.017	−16.144 ± 17.092	−2.256 ± 24.431 b,c
Δ HF (n.u)	24.816 ± 17.708	19.190 ± 15.908	7.223 ± 17.296 b,c
Δ LF/HF	−4.038 ± 3.343	−3.854 ± 5.929	0.337 ± 5.201 b,c

Comparisons between groups: b (*p* < 0.05) healthy valve vs. aortic valve stenosis, c (*p* < 0.05) aortosclerosis vs. aortic valve stenosis. Comparisons between supine position and active standing: * (*p* < 0.05), ** (*p* < 0.001). Mean NN = mean value of all NN intervals. SDNN = standard deviation of all NN intervals. SDSD = root mean square of the successive differences. pNN20: percentage of successive NN intervals with differences greater than 20 ms. LF n.u = low-frequency band (normalized units); HF n.u = high-frequency band (normalized units). LF/HF = ratio between low-frequency and high-frequency band indices. Δ = difference between the values in the supine position, and the values after active standing in each HRV index.

**Table 3:** Pearson’s correlation coefficients between meanNN and linear heart rate variability indices. The estimation of each correlation coefficient includes data obtained at both the supine position and during active standing.

	Healthy Valve (N = 22)	Aortic Valve Sclerosis (N = 73)	Aortic Valve Stenosis (N = 32)	Total (N = 127)
**Statistical indices**				
SDNN (s)	0.611 **	0.394 **	0.292 *	0.404 **
pNN20 (%)	0.830 **	0.602 **	0.686 **	0.635 **
SDSD (s)	0.832 **	0.595 **	0.643 **	0.638 **
**Spectral indices**				
LF n.u	−0.487 **	−0.312 **	−0.362 *	−0.349 **
HF n.u	0.486 **	0.363 **	0.396 *	0.389 **
LF/HF	−0.490 **	−0.264 **	−0.167	−0.255 **

* (*p* < 0.05), ** (*p* < 0.001).

**Table 4:** Pearson’s correlation coefficients between meanNN and nonlinear indices of heart rate variability. The estimation of each correlation coefficient (r) includes data obtained at both the supine position and during active standing.

	Healthy Valve (N = 22)	Aortic Valve Sclerosis (N = 73)	Aortic Valve Stenosis (N = 32)	Total (N = 127)
Determinism	−0.742 **	−0.562 **	−0.426 **	−0.554 **
Mean diagonal length	−0.641 **	−0.354 **	−0.304 *	−0.329 **
Maximum diagonal length	−0.550 **	−0.507 **	−0.341 *	−0.446 **
Shannon entropy	−0.716 **	−0.446 **	−0.412 **	−0.450 **
Laminarity	−0.765 **	−0.554 **	−0.463 **	−0.564 **
Trapping Time	−0.660 **	−0.443 **	−0.449 **	−0.448 **
Maximum vertical length	−0.648 **	−0.615 **	−0.607 **	−0.600 **
TT 1	−0.029	0.112	0.072	0.073
TT 2	−0.703 **	−0.436 **	−0.370 *	−0.449 **

* (*p* < 0.05), ** (*p* < 0.001).

**Table 5:** Linear stepwise multiple regression analysis with predicted HRV linear indices and independent variables: meanNN (s), the aortic valve stenosis (AVS) condition (dichotomized between HV and AVD), systolic blood pressure (SBP, mmHg), and age (years).

Variables	Standardized β	β (C.I.95%)	*p*-Value	Corrected R^2^
Predicted HRV index: SDNN				0.157
Mean NN	0.353	0.059 (0.029–0.089)	<0.001	
AVS condition		Excluded variable		
Age		Excluded variable		
SBP		Excluded variable		
Statins		Excluded variable		
Predicted HRV index: pNN20				0.509
Mean NN	0.571	81.213 (61.857–100.569)	<0.001	
AVS condition		Excluded variable		
Age	−0.351	−0.642 [−1.014–(−0.270)]	0.001	
SBP		Excluded variable		
Statins		Excluded variable		
Predicted HRV index: SDSD				0.439
Mean NN	0.586	0.081 (0.061–0.101)	<0.001	
AVS condition		Excluded variable		
Age	−0.294	−0.001 (−0.001–0.000)	0.008	
SBP		Excluded variable		
Statins		Excluded variable		
Predicted HRV index: LFnu.				0.148
Mean NN	−0.210	−30.794 [−57.072–(−4.517)]	0.022	
AVS condition		Excluded variable		
Age		Excluded variable		
SBP	0.348	0.409 (0.165–0.652)	0.001	
Statins		Excluded variable		
Predicted HRV index: HFnu.				0.148
Mean NN	0.206	24.591 (3.146–46.036)	0.025	
AVS condition		Excluded variable		
Age		Excluded variable		
SBP	−0.308	−0.294 [−0.493–(−0.096)]	0.004	
Statins		Excluded variable		
Predicted HRV index: LF/HF				0.099
Mean NN		Excluded variable		
AVS condition		Excluded variable		
Age		Excluded variable		
SBP	0.243	0.066 (0.008–0.124)	0.026	
Statins	0.246	5.455 (1.016–9.893)	0.017	

**Table 6:** Linear stepwise multiple regression analysis with predicted HRV nonlinear indices and independent variables: meanNN (s), the aortic valve stenosis (AVS) condition (dichotomized between supine position and active standing position), systolic blood pressure (SBP, mmHg), and age (years).

Variables	Standardized β	β (C.I.95%)	*p*-Value	Corrected R^2^
Predicted HRV index: Determinism				0.313
Mean NN	−0.409	−0.462 [−0.644–(−0.280)]	<0.001	
AVS condition		Excluded variable		
Age		Excluded variable		
SBP	0.303	0.003 (0.001–0.004)	0.002	
Statins	0.245	0.180 (0.051–0.308)	0.006	
Predicted HRV index: Mean diagonal length				0.187
Mean NN	−0.236	−0.873 [−1.521–(−0.226)]	0.009	
AVS condition		Excluded variable		
Age		Excluded variable		
SBP	0.326	0.010 (0.004–0.016)	0.002	
Statins		Excluded variable		
Predicted HRV index: Maximum diagonal length				0.283
Mean NN	−0.315	−24.024 [−36.549–(−11.500)]	<0.001	
AVS condition		Excluded variable		
Age		Excluded variable		
SBP	0.378	0.230 (0.114–0.346)	<0.001	
Statins	0.185	9.172 (0.329–18.016)	0.042	
Predicted HRV index: Shannon entropy				0.278
Mean NN	−0.344	−0.762 [−1.128–(−0.397)]	<0.001	
AVS condition		Excluded variable		
Age		Excluded variable		
SBP	0.295	0.005 (0.002–0.009)	0.003	
Statins		Excluded variable		
Predicted HRV index: Laminarity				0.293
Mean NN	−0.434	−0.583 [−0.802–(−0.364)]	<0.001	
AVS condition		Excluded variable		
Age		Excluded variable		
SBP	0.226	0.002 (0.000–0.004)	0.019	
Statins	0.247	0.216 (0.061–0.370)	0.007	
Predicted HRV index: Trapping Time				0.302
Mean NN	−0.321	−0.968 [−1.457–(−0.479)]	<0.001	
AVS condition		Excluded variable		
Age		Excluded variable		
SBP	0.291	0.007 (0.002–0.012)	0.003	
Statins		Excluded variable		
Predicted HRV index: Maximum vertical length				0.391
Mean NN	−0.566	−12.133 [−15.379–(−8.886)]	<0.001	
AVS condition		Excluded variable		
Age		Excluded variable		
SBP	0.226	0.039 (0.009–0.069)	0.012	
Statins		Excluded variable		
Predicted HRV index: TT 1				0.047
Mean NN		Excluded variable		
AVS condition		Excluded variable		
Age		Excluded variable		
SBP	−0.238	−0.021 [−0.040–(−0.002)]	0.034	
Statins		Excluded variable		
Predicted HRV index: TT 2				0.157
Mean NN	−0.263	−12.750 [−21.388–(−4.113)]	0.004	
AVS condition		Excluded variable		
Age		Excluded variable		
SBP		Excluded variable		
Statins	0.247	7.768 (1.668–13.867)	0.013	

**Table 7:** Graphic representation of comparisons of the behavior of HRV RQA indices in AVD and orthostatic challenge, shown as arrows in direction of change *.

	Supine Position vs. Active Standing (Within Same Group)			Comparisons vs. Healthy Valve (During Supine Position)	
HRV Nonlinear Indices	Healthy Valve	Aortic Valve Sclerosis	Aortic Valve Stenosis	Aortic Valve Sclerosis	Aortic Valve Stenosis
Determinism	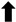	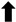	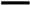	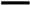	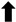
Mean diagonal length	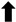	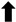	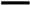	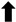	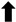
Maximum diagonal length	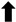	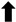	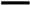	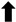	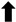
Shannon entropy	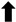	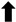	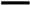	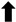	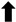
Laminarity	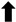	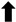	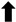	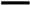	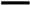
Trapping Time	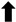	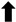	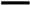	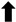	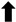
Maximum vertical length	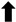	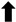	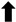	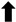	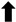
Trapping Time 1	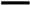	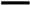	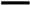	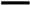	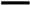
Trapping Time 2	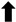	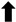	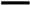	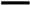	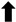

* Statistically significant comparisons *p* < 0.05 between the indices were represented by arrows, so an upward arrow = increase in index value, dash there were no statistically significant changes.

**Table 8:** Graphic representations of comparisons of the Δ values of HRV RQA indices in AVD and orthostatic challenge are shown as arrows in the direction of change, as well as deltas different from zero.

	Δ Different from Zero			Comparisons vs. Healthy Valve (During Supine Position)	
Δ HRV Nonlinear Indices	Healthy Valve	Aortic Valve Sclerosis	Aortic Valve Stenosis	Aortic Valve Sclerosis	Aortic Valve Stenosis
Δ Determinism	*	*	**-**	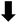	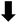
Δ Mean diagonal length	*	*	**-**	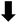	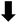
Δ Maximum diagonal length	*	*	**-**	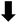	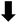
Δ Shannon entropy	*	*	**-**	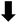	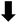
Δ Laminarity	*	*	**-**	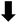	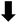
Δ Trapping Time	*	*	**-**	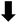	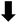
Δ Maximum vertical length	*	*	*	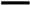	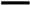
Δ Trapping Time 1	**-**	**-**	**-**	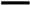	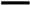
Δ Trapping Time 2	*	*	**-**	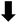	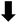

Hence * = Δ different from zero, - = deltas with value of zero. Statistically significant comparisons *p* < 0.05 between the indices were represented by arrows, downward arrow = decrease in index value, dash line indicates that there were no statistically significant changes.

## Data Availability

The raw data supporting this article’s conclusions will be made available upon request to the corresponding author, provided pertinent legal requirements are met.

## Supplementary Materials

The following supporting information can be downloaded at: https://www.mdpi.com/article/10.3390/s25051535/s1, Table S1. Results of the analysis of recurrence rates of heart rate variability. Data are shown as mean ± standard deviation or median (25th percentile–75th percentile). Table S2. Graphic representation of comparisons of the behavior of HRV linear indices in AVD and orthostatic challenge, shown as arrows in direction of change. Table S3. Graphic representation of comparisons of the magnitude of change of HRV linear indices in AVD in response to the orthostatic challenge.
