# Supplementary material for: Dynamic Response of Heart Rate Variability to Active Standing in Aortic Valve Disease: Insights from Recurrence Quantification Analysis
Source: Sensors (Basel). 2025 Mar 1;25(5):1535. doi: 10.3390/s25051535 (PMC11902333; doi:10.3390/s25051535)
Supplement: Supplementary file 1 [file sensors-25-01535-s001.zip › sensors-3412959-supplementary.pdf]

Supplementary Materials

Table S1. Results of the analysis of recurrence rates of heart rate variability. Data are shown as mean ± standard deviation or median (25th percentile – 75th percentile).

	Healthy valve (N = 22)	Aortic valve sclerosis (N = 73)	Aortic valve stenosis (N = 32)
**Supine Position**			
Determinism	0.319 ± 0.107 **<br>0.306 (0.252 – 0.372)	0.388 ± 0.161**<br>0.370 (0.255 – 0.463)	0.450 ± 0.195 b<br>0.440 (0.306 – 0.597) b
Mean diagonal length	2.323 (2.270 - 2.410) **	2.367 (2.264 - 2.545) **	2.541 (2.384 - 2.918) b,c
Maximum diagonal length	10.5 (7 – 14) *	12 (8 – 19) **	16 (12 - 25.5) b,c
Shannon entropy	0.717 (0.655 - 0.802) **	0.767 (0.605 - 0.939) **	0.959 (0.771 - 1.244) b,c
Laminarity	0.401 ± 0.141 **<br>0.370 (0.332 – 0.502)	0.46 ± 0.197 **<br>0.471 (0.326 – 0.604)	0.516 ± 0.252 *<br>0.582 (0.409 – 0.700)
Trapping Time	2.222 (2.155 - 2.401) **	2.346 (2.231 - 2.64) a**	2.824 (2.537 - 3.163) b,c
Maximum vertical length	7 (5 – 8) **	8 (6 – 9) **	9 (7 - 11.5) b,c *
Trapping Time 1	14.381 (13.982 -15.190)	14.595 (13.931 - 15.375)	15.089 (14.255 - 15.78)
Trapping Time 2	19.250 (17.960 - 20.377)**	20.024 (18.419 - 24.988)**	22.785 (18.252 - 29.460) b
**Active Standing**			
Determinism	0.544 ± 0.150<br>0.560 (0.440 -0.682)	0.568 ± 0.150<br>0.608 (0.463 – 0.679)	0.49 ± 0.179<br>0.505 (0.345 – 0.596)
Mean diagonal length	2.571 (2.366 - 2.858)	2.686 (2.433 - 2.952)	2.550 (2.388 - 3.039)
Maximum diagonal length	19 (14 – 30)	21 (16 – 25)	19.5 (10 – 27)
Shannon entropy	0.998 (0.779 - 1.219)	1.092 (0.86 - 1.294)	0.979 (0.766 - 1.314)
Laminarity	0.666 ± 0.135<br>0.688 (0.605 – 0.791)	0.674 ± 149<br>0.717 (0.594 – 0.774)	0.592 ± 0.177 c<br>0.612 (0.499 – 0.729)c
Trapping Time	2.801 (2.627 - 3.089)	2.912 (2.552 - 3.216)	2.850 (2.541 - 3.317)
Maximum vertical length	10 (9 – 12)	12 (9 – 14)	10 (8 - 13.5)
Trapping Time 1	14.773 (14.305 - 15.239)	15.010 (14.416 - 15.418)	14.704 (14.180 - 15.200)
Trapping Time 2	27.000 (23.538 - 32.618)	28.701 (24.329 - 33.567)	26.508 (22.056 - 30.085)
**Magnitude of change**			
Δ Determinism	-0.224 ± 0.148<br>-0.197 [-0.365 – (-0.078)]	-0.180 ± 0.167<br>-0.192 [-0.283 – (-0.064)]	-0.039 ± 0.165b,c<br>-0.016 (-0.143 – 0.083)b
Δ Mean diagonal length	-0.235 [-0.514-(-0.074)]	-0.182 [-0.488-(-0.023)]	0.001 (-0.228-0.3394)b, c
Δ Maximum diagonal length	-9.500 [-17.500-(-4.250)]	-6.00(-12.000-0.000)	-2.000 (-7.000-9.000)b,c
Δ Shannon entropy	-0.274 [-0.485-(-0.083)]	-0.237 [-0.504-(-0.064)]	-0.019 (-0.211-0.246)b,c
Δ Laminarity	-0.265 ± 0.159<br>-0.257 [-0.369 – (- 0.115)]	-0.207 ± 0.175<br>-0.227 [-0.311 – (-0.087)	-0.076 ± 0.195 b,c<br>-0.037 (-0.156 – 0.074)b,c
Δ Trapping Time	-0.499 [-0.785-(-0.294)]	-0.396 [-0.788-(-0.194)]	-0.1603 (-0.346-0.137)b,c
Δ Maximum vertical length	-3.000 [-6.250-(-1.000)]	-4.000 [-6.000-(-20-.000)]	-2.000 [-4.750-(-0.250)]
Δ Trapping Time 1	-0.096 (-0.898-0.624)	-0.150 (-1.121-0.647)	0.239 (-0.815-1.407)
Δ Trapping Time 2	-7.241 [-13.323-(-2.304)]	-7.410 [-11.450-(-2.793)]	-1.237 (-6.294-2.357)b,c

Comparisons between groups: b (p<0.05) Healthy valve vs. Aortic valve stenosis, c (p<0.05) Healthy valve vs Aortic valve sclerosis * (p< 0.05), **(p< 0.001) supine position vs active standing (same group). Determinism =the percentage of recurrence points forming diagonals from all recurrence points. Shannon entropy= the probability of finding a diagonal line. Laminarity = proportion of recurrence points forming vertical lines. Trapping time =

time in which the dynamics remain trapped in a certain state. Δ = difference between the values in supine position and active standing in each HRV index.

Table S2. Graphic representation of comparisons of the behavior of HRV linear indices in AVD and orthostatic challenge, shown as arrows in direction of change.

**HRV linear indices**	**Supine position vs active standing (within same group)**			**Comparisons vs Healthy valve (during supine position)**	
	**Healthy valve**	**Aortic valve sclerosis**	**Aortic valve stenosis**	**Aortic valve sclerosis**	**Aortic valve stenosis**
Mean NN (s)	↓	↓	↓	—	—
SDNN (s)	—	↓	—	—	—
pNN20 (%)	↓	↓	↓	↓	↓
SDSD (s)	↓	↓	↓	—	—
LF n.u	↑	↑	—	—	↑
HF n.u	↓	↓	↓	—	↓
LF/HF	↑	↑	—	—	↓

Statistically significant comparisons $p<0.05$ between the indices were represented by arrows, so up arrow = increase in index value, down arrow = decrease in index value, dash there were no statistically significant changes.

Table S3. Graphic representation of comparisons of the magnitude of change of HRV linear indices in AVD and orthostatic challenge, shown as arrows in direction of change. As well as, deltas different from zero.

**Δ HRV linear indices**	**Δ different from zero**			**Comparisons vs Healthy valve (during supine position)**	
	**Healthy valve**	**Aortic valve sclerosis**	**Aortic valve stenosis**	**Aortic valve sclerosis**	**Aortic valve stenosis**
Δ Mean NN (s)	*	*	*	↓	↓
Δ SDNN (s)	-	*	-	—	—
Δ pNN20 (%)	*	*	*	↓	—
Δ SDSD (s)	*	*	*	—	—
Δ LF n.u	*	*	-	↓	↓
Δ HF n.u	*	*	*	↓	↓
Δ LF/HF	*	*	-	↓	↓

Δ different from zero determined from the Wilcoxon test with test value 0. Hence * = deltas different from zero, - = deltas with value of zero. Statistically significant comparisons $p<0.05$ between the indices were represented by arrows, so up arrow = increase in index value, down arrow = decrease in index value, dash there were no statistically significant changes.
